# Association of Behçet’s disease with the risk of metabolic syndrome and its components: a systematic review and meta-analysis

**DOI:** 10.1007/s10238-023-01044-x

**Published:** 2023-03-20

**Authors:** Tingqiao Chen, Xinyi Shao, Hao Li, Yangmei Chen, Lin Liu, Judan Zhong, Jin Chen

**Affiliations:** 1 Department of Dermatology, The First Affiliated Hospital of Chongqing Medical University, Chongqing, 400016 China; 2 Department of Dermatology, Children’s Hospital of Chongqing Medical University, National Clinical Research Center for Child Health and Disorders, Ministry of Education Key Laboratory of Child Development and Disorders, Chongqing, China

**Keywords:** Behçet’s disease, Metabolic syndrome, Meta-analysis, Diabetes mellitus, Hypertension, Dyslipidemia

## Abstract

The present meta-analysis aimed to elucidate the association of Behçet’s disease (BD) with the risk of metabolic syndrome (MetS) and its components. Observational cohort studies were searched from the Embase, Web of Science, Medline, and Cochrane Library databases. The primary outcome was the association of BD with the risk of MetS and its relevant components. Effect estimates with odds ratios (ORs) were pooled using either the random-effects or fixed-effects models, according to heterogeneity. Leave-one-out sensitivity analyses were used to determine the stability of the results. Twenty-three studies, comprising 42,834 patients with BD, were included. Overall, a significant association between BD and the risk of MetS was found (pooled OR 2.26; 95% confidence interval [CI] 1.61–3.17; *P* < 0.0001). Among the components of MetS, significant associations were found between BD and diabetes mellitus (OR 1.21; 95% CI 1.10–1.33; *P* < 0.0001), BD and hypertension (OR 1.39; 95% CI 1.13–1.70; *P* = 0.002), and BD and dyslipidemia (OR 1.21; 95% CI 1.01–1.45; *P* = 0.04). Our study indicated an association between BD and the risk of MetS and some of its components (diabetes mellitus, hypertension, and dyslipidemia). Physician should consider these associations so that specific treatments are available for patients with comorbidities. Moreover, patients with BD should regularly monitor their blood pressure, fasting plasma glucose, and blood lipid levels.

**Supplementary Information:**

The online version contains supplementary material available at 10.1007/s10238-023-01044-x.

## Introduction

Behçet’s disease (BD) is a multisystemic autoimmune and chronic inflammatory vasculitis, characterized by recurrent painful mouth sores, genital ulcers, uveitis, skin lesions, and other systemic manifestations [1]. It frequently occurs in patients originating in the Middle East, Far East, and Mediterranean, and is also known as Silk Route disease. The reported prevalence of BD in East Asia ranges from 13.5 to 27 per 100,000 persons [2, 3]. BD has significant morbidity and mortality risks, with the main causes of death being ruptured coronary/pulmonary arterial aneurysms, neurological involvement, and thrombosis [4–8]. Although genetic susceptibility, inflammation, and immunological abnormalities have been verified to play decisive roles in BD progression, the pathological mechanism of BD is not completely understood [9]. As research on BD has increased in recent years, it is vital to investigate its comorbidities.

Metabolic syndrome (MetS) affects 14–32% of the world’s population, and its incidence continues to increase [10]. MetS is a set of metabolic abnormalities that includes hypertension, glucose intolerance, abdominal obesity, and atherogenic dyslipidemia, thereby increasing the risk of cardiovascular disease and mortality [11]. The cumulative effects of longstanding inflammation resulting from chronic inflammatory diseases are major contributing factors to MetS. Furthermore, numerous studies have confirmed a link between MetS and inflammatory diseases such as psoriasis and hidradenitis suppurativa [12, 13].

Recent studies have reported that patients with BD are more likely to have MetS than health control (HCs) [14–16]; however, results of studies evaluating the relationship between BD and the risk of MetS and its components remain inconsistent. Therefore, this study was performed to elucidate the relationship of BD and the risk of MetS and its components.

## Methods

This study adhered to the Meta-analysis of Observational Studies in Epidemiology (MOOSE) and the Preferred Reporting Items for Systematic Review and Meta-Analyses (PRISMA) (http://www.prisma-statement.org/) guidelines [17, 18].

### Data sources and searches

Two independent authors (TC and XS) searched for studies published before November 31, 2022, using electronic databases (Embase, Web of Science, MEDLINE, and Cochrane Library). The terms used included Behçet syndrome, Behçet disease, Behçet’s syndrome, Silk syndrome, Behçets syndrome, Behçet’s disease, BD, metabolic syndrome, metabolic disorders, hypertension, blood pressure, fasting blood glucose, plasma glucose, dyslipidemia, triglyceride, HDL, waist circumference, obesity, and abdominal obesity. We also performed a manual supplemental search by reviewing the reference lists of relevant articles, systematic reviews, and meta-analyses to avoid potentially missing articles. The review protocol was registered in the International Prospective Register of Systematic Reviews (PROSPERO) (CRD42022344815).

### Eligibility criteria for selecting studies

The inclusion criteria for studies in the meta-analysis were as follows: (1) prospective or retrospective observational studies; (2) studies involving human participants; (3) studies investigating the association of BD with MetS or its relevant components, and studies describing the prevalence of MetS or its relevant components in BD patients; and (4) studies published in English.

### Types of outcome measures

The primary outcome was the association between BD and the risk of MetS and its relevant components.

### Data extraction and quality assessment

Two authors (TC and XS) independently engaged in the study selection, data collection, and extraction. In the case of incomplete data, we emailed the authors to obtain supplementary information. The quality of each eligible study was assessed using the Newcastle–Ottawa scale (NOS) by each investigator to evaluate the quality [19]. The NOS awards a maximum of nine points for each study and is based on three major components: selection of the groups, comparability, and exposure; a score of 7–9 indicates high quality (low risk of bias). Any disagreements were resolved by consensus and included a third author (HL).

### Statistical analysis

All statistical analyses were performed using Review Manager 5.4 software (The Nordic Cochrane Center, Copenhagen, Denmark) and the Bioconductor programming environment 22 (R, version 4.2.1). Pooled odds ratios (ORs) with 95% confidence intervals (CIs) were calculated to assess the prevalence of MetS and its components in the comparison between patients with BD and controls. Statistical heterogeneity between studies was calculated using the *I*^2^ test; *I*^2^ > 50% indicated that the studies were heterogeneous. If considerable heterogeneity (*I*^2^ > 50%) was recognized, the random-effects model was employed (DerSimonian and Laird method); otherwise, a fixed-effects model was used (Mantel–Haenszel method). Begg’s test was conducted to evaluate publication bias [20, 21].

## Results

### Description of included studies

**Fig. 1:**
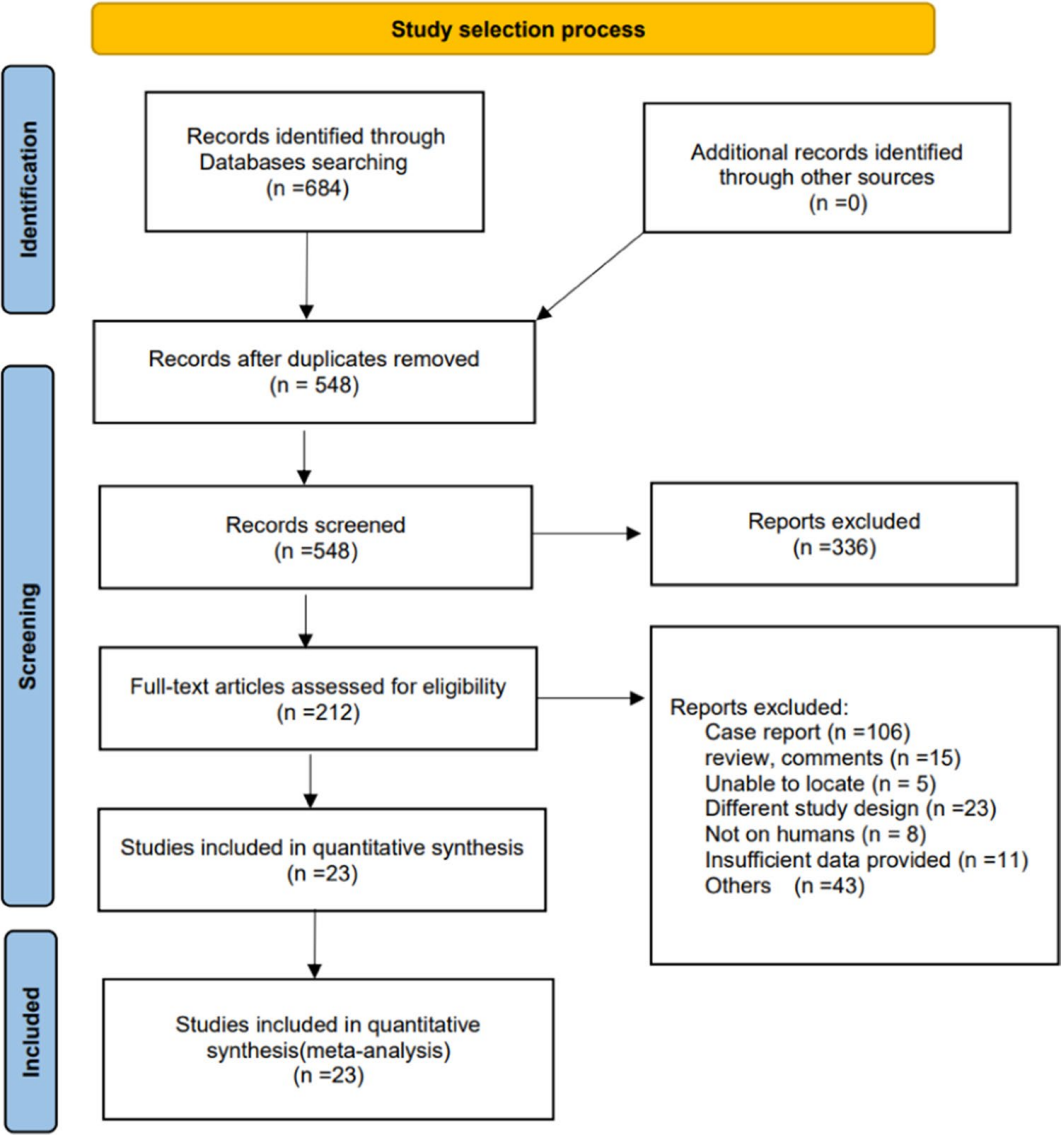
Selection process for eligible studies included in the systematic review and meta-analysis

The literature search process is illustrated in Fig. 1. We included 23 studies with 684 records identified through computerized database searches [3, 14, 22–42]; the characteristics of the selected 23 studies are shown in Table 1. The 23 eligible studies, published between 2005 and 2022, comprised 42,834 patients with BD and 26,977 controls. There were five cross-sectional and 18 case–control studies, among which three were from Africa, one from the United States of America, one from Europe, and the remainder from Asia. The quality assessment scores obtained using the NOS for the eligible studies are summarized in Table 2.

**Table 1:** Characteristics of the 23 selected studies

References	Enthnicity	Type of study	Group	Number (*n*)	Age (mean ± SD, or range, or median [IQR]) (years)	Sex (M/F)(n)	Clinical signs			
							MetS (*n*)	Obesity (*n*)	BMI (kg/m^2^)	Waistline (cm)
Erdem et al. [29]	Asian	Case–control	BD	14	23.86 ± 0.89	/			21.64 ± 0.82	
			NBD	15	24.13 ± 0.59	/			23.00 ± 0.53	
Ugurlu et al. [36]	Asian	Case–control	BD	225	52 ± 8	141/84			27.4 ± 4.3	
			NBD	117	50 ± 5	74/43			26.6 ± 4.4	
Kim et al. [24]	Asian	Case–control	BD	82	43.9 ± 11.4	26/56	5		22.9 ± 3.5	82.4 ± 9.0
			NBD	89	44.1 ± 9.1	40/49	8		23.6 ± 3.2	80.7 ± 9.4
Messedi et al. [33]	African	Case–control	BD	50	48 (41–54)	35/15				88.08 ± 8.6
			NBD	50	46 (40–54)	35/15				91.10 ± 10.11
Ulaşoğlu [27]	Asian	Case–control	BD	115	47.6 ± 9.1	48/67	62		28.4 ± 5.0	103.8 ± 12.3
			NBD	65	42.7 ± 14.5	27/38	18		23.7 ± 4.9	100.6 ± 11.9
Ricart et al. [35]	European	Case–control	BD	89	44 ± 12	48/41		13	25.3 ± 4.3	
			NBD	89	43 ± 10	47/42		6	25.1 ± 3.4	
Yalçın et al. [14]	Asian	Case–control	BD	86	39.05 ± 10.1	32/54	30			
			NBD	72	38.96 ± 11.4	23/49	14			
Erden et al. [26]	Asian	Case–control	BD	25	33.24 ± 7.18	13/12	7		23.92 ± 1.5	
			NBD	25	34.12 ± 5.74	13/12	4		23.51 ± 1.32	
Pandey et al. [42]	America	Cross-sectional	BD	2540	43.9 (0.63)	642/1898				
Gul et al. [22]	Asian	Case–control	BD	30	35.10 ± 7.35	15/15	5		23.59 ± 1.52	
			NBD	30	31.99 ± 6.97	15/15	3		24.32 ± 2.47	
Erden et al. [25]	Asian	Case–control	BD	30	36.66 ± 7.9	15/15	12		24.08 ± 1.57	85.53 ± 8.37
			NBD	30	37.20 ± 11.3	15/15	12		24.47 ± 1.63	87.33 ± 9.58
El-Gazzar et al. [23]	Asian	Case–control	BD	38	36.2 ± 7.8	8/30	11		26.9 ± 3.9	
			NBD	38	35.4 ± 6.5	8/30	4		25.4 ± 3.1	
Yavne et al. [37]	Asian	Case–control	BD	871	49.0 ± 15.5	458/413				
			NBD	4349	49.8 ± 15.4	2288/2061				
Koca et al. [31]	Asian	Case–control	BD	143	37.7 ± 10.9	61/82		18	25.3 ± 4.8	
			NBD	112	40.1 ± 13.9	48/64		23	26.3 ± 5.5	
Acikgoz et al. [34]	Asian	Case–control	BD	60	44.1 ± 8.3	29/31			23.0 ± 1.6	
			NBD	50	45.4 ± 7.4	27/33			22.9 ± 1.5	
Lee et al. [3]	Asian	Cohort study	BD	19,937	/	6502/13435				
Gheita et al. [41]	African	Cross-sectional	BD	1526	35.7 ± 9.84	1102/424			27.57 ± 5.24	
Chen et al. [39]	Asian	Cohort study	BD	6508	38.1 ± 15.1	2837/3671				
Lee et al. [40]	Asian	Cohort study	BD	6178	/	/	1609			
Lin et al. [32]	Asian	Case–control	BD	1554	39.2 ± 12.0	653/901				
			NBD	3108	39.1 ± 12.2	1373/1735				
Cebeci Kahraman [28]	Asian	Case–control	BD	60	34.03 ± 8.05	44/16			24.41 ± 3.06	
			NBD	45	30.87 ± 8.4	25/20			24.31 ± 2.85	
Jung et al. [30]	Asian	Case–control	BD	6214	46.9 ± 13.2	7077/11565				85.72 ± 8.4
			NBD	18,642	46.9 ± 13.2	2359/3855				82.84 ± 9.8
ElAdle et al. [38]	African	Case–control	BD	1028	36.8 ± 10.1	750/278	234		28.6 ± 5.8	89.7 ± 14.7
			NBD	51	34.3 ± 10.9	42/9	3		28.7 ± 5.4	90.5 ± 12.8

**Table 2:** Quality assessment scores (NOS scale tool) for the eligible studies NOS, Newcastle–Ottawa scale

	Selection				Comparability		Exposure			Scores
	Cases definition	Cases representativeness	Controls selection	Controls definition	Age matching	Additional matching	Ascentaiment exposure	Same method for cases and controls	Non-response rate	
Erdem et al. [29]	*		*	*	*	*	*	*	*	8
Ugurlu et al. [36]	*	*		*		*	*	*	*	7
Kim et al. [24]	*		*	*	*	*	*	*	*	8
Messedi et al. [33]	*		*	*	*	*	*	*	*	8
Ricart et al. [35]	*			*	*		*	*	*	6
Ulaşoğlu [27]	*		*	*	*		*	*	*	7
Yalçın et al. [14]	*	*	*	*	*	*	*	*	*	9
Erden et al. [26]	*		*	*			*	*	*	6
Pandey et al. [42]	*	*	*	*	*		*	*	*	8
Gul et al. [22]	*		*	*	*	*	*	*	*	8
El-Gazzar [23]	*		*	*	*	*	*	*	*	8
Erden et al. [25]	*		*	*	*	*	*	*	*	8
Koca et al. [31]	*			*	*	*	*	*	*	7
Yavne et al. [37]	*	*	*	*	*	*	*	*	*	9
Acikgoz et al. [34]	*	*		*	*	*	*	*	*	8
Lee et al. [3]	*	*	*	*		*	*	*	*	8
Gheita et al. [41]	*	*	*	*	*		*	*	*	8
Chen et al. [39]	*	*	*	*	*	*	*	*	*	9
Lin et al. [32]	*	*	*	*	*	*	*	*	*	9
Lee et al. [40]	*	*	*	*		*	*	*	*	8
Cebeci Kahraman [28]	*			*	*	*	*	*	*	7
Jung et al. [30]	*	*	*	*	*	*	*	*	*	9
ElAdle et al. [38]	*	*	*	*	*	*	*	*	*	9

### Behcet’s disease and metabolic syndrome

**Fig. 2:**
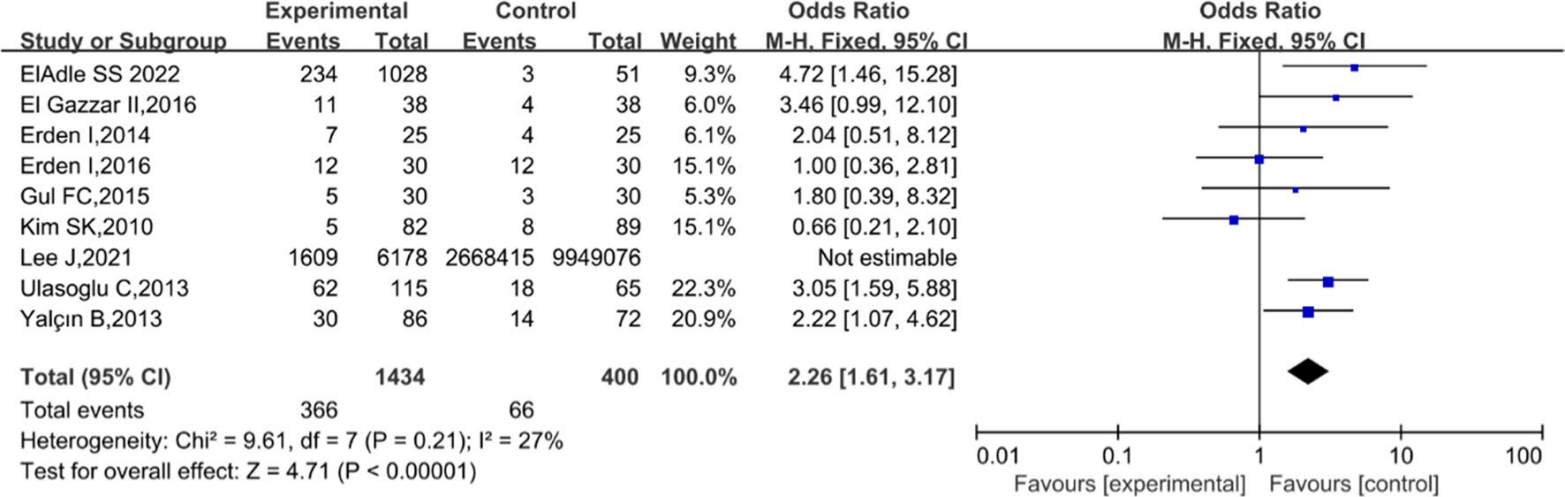
Association between Behçet’s disease and metabolic syndrome. Forest plot for the association between Behçet’s disease and metabolic syndrome

The prevalence of MetS in patients with BD was reported in eight studies [14, 22–27]. Among the included studies, MetS was reported in 25.52% of patients with BD (366/1434), whereas only 16.50% were found to be affected by MetS in the non-BD group (66/400). The pooled analysis showed that BD was significantly associated with MetS (OR 2.26; 95% CI 1.61–3.17; *P* < 0.0001; Fig. 2). Moreover, the fixed-effects model was used because eligible trials demonstrated low heterogeneity (*I*^2^ = 27%; *P* = 0.21). Additionally, we did not examine publication bias based on the symmetry of the funnel plot and the Begg’s test (*P* = 0.4579).

### Behcet’s disease and diabetes mellitus

**Fig. 3:**
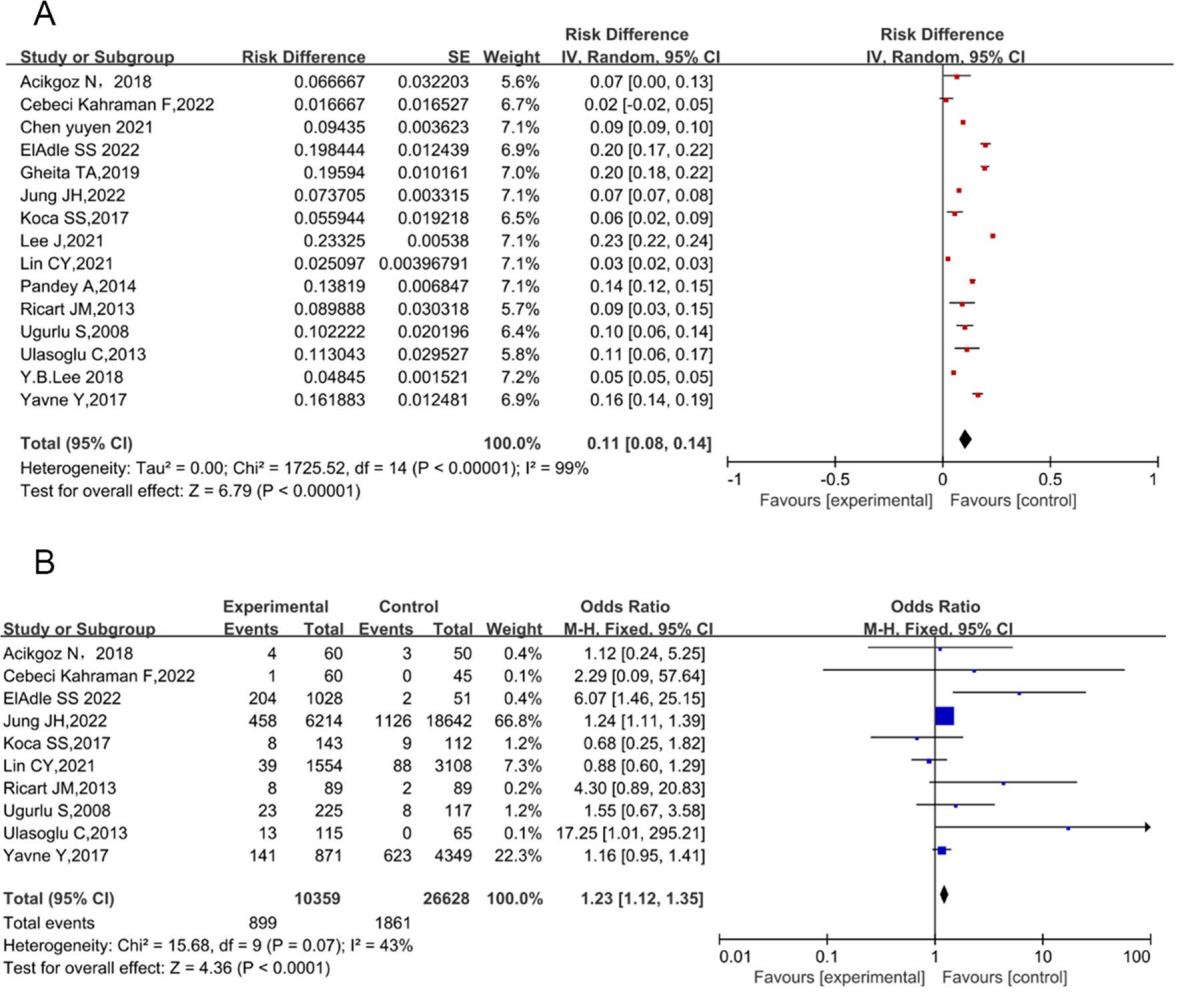
Forest plots for the association between Behçet’s disease and diabetes mellitus. Observational studies of the association between Behçet’s disease and diabetes mellitus (**A**). Case–control studies of the association between Behçet’s disease and diabetes mellitus (**B**)

Fifteen studies reported the prevalence of diabetes mellitus in patients with BD, including 10 case–control studies, three cohort studies, and two cross-sectional studies. The pooled prevalence of diabetes mellitus in patients with BD was 11% (95% CI 8% to 14%; *P* < 0.0001), and the heterogeneity of these studies was evident (*I*^2^ = 99%; *P* = 0.000; Fig. 3A). Excluding five studies that did not report the number of patients with diabetes mellitus among non-BD participants, pooled analysis of the other 10 case–control studies [27, 28, 30–32, 34–38] showed that diabetes mellitus was detected in 7.45% of patients with BD, and 7.00% of controls. The association between BD and diabetes mellitus was considered significant (OR 1.23; 95% CI 1.12–1.35; *P* < 0.0001; Fig. 3B), with low heterogeneity (*I*^2^ = 43%; *P* = 0.07). The prevalence of diabetes mellitus in patients with BD using the Begg’s test (*P* = 0.3252) revealed no publication bias. Moreover, seven studies that reported fasting blood glucose levels in patients with BD were included, and no significant difference in fasting blood glucose was observed (mean difference [MD], 1.00; 95% CI −3.12–5.11; *P* = 0.64; Supplementary Fig. 1) [22–24, 26, 28, 34, 35].

### Behcet’s disease and dyslipidemia

**Fig. 4:**
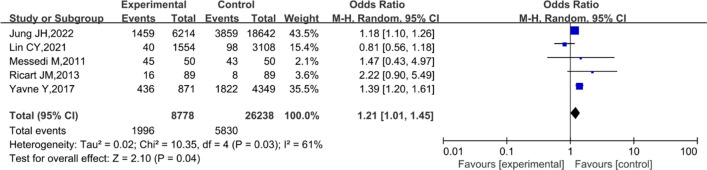
Forest plot for the association between Behçet’s disease and dyslipidemia

As illustrated in Fig. 4, the prevalence of dyslipidemia was significantly associated with BD (OR 1.21; 95% CI 1.01–1.45; *P* = 0.04; Fig. 4), with a substantial heterogeneity (*I*^2^ = 61%; *P* = 0.03) [30, 32, 33, 35, 37]. In addition, the sensitivity analysis showed that the overall statistical significance of the meta-analysis did not change after removal of any individual study, indicating that the results were stable and credible. Moreover, a non-significant publication bias was reported based on the results of the Begg’s test (*P* = 1.0000).

Twelve studies recorded triglyceride levels [22–29, 31, 34, 35, 38], and nine studies recorded high-density lipoprotein (HDL) levels [22–28, 34, 38] in patients with BD. The meta-analysis illustrated that elevated triglyceride levels were not significantly related to BD (MD, 9.36; 95% CI −1.96–20.68; *P* = 0.11; Supplementary Fig. 2). A lower HDL level was observed in patients with BD in most studies; however, the difference was not markedly significant (MD, −2.28; 95% CI −5.38–0.81; *P* = 0.15; Supplementary Fig. 3).

### Behcet’s disease and hypertension

**Fig. 5:**
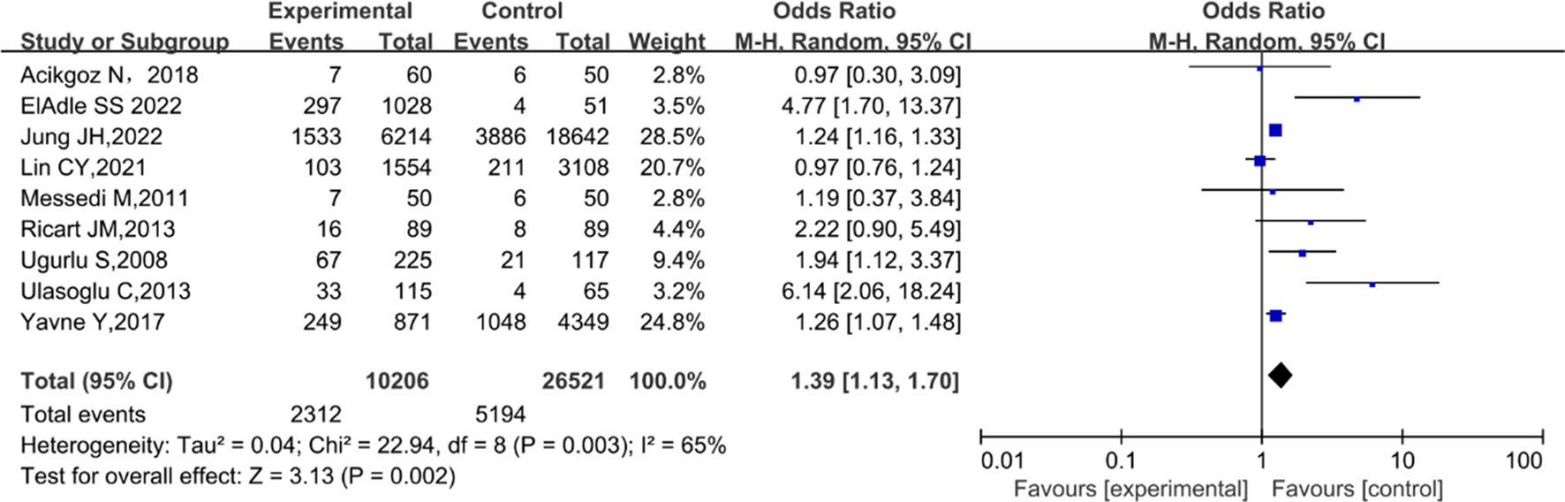
Forest plot for the association between Behçet’s disease and hypertension

The results of the nine trials including 35,648 patients, also demonstrated an increased OR for hypertension in association with BD [27, 30, 32–38]. The meta-analysis revealed a significant association between BD and hypertension (pooled OR 1.39; 95% CI 1.13–1.70; *P* = 0.002), with considerable heterogeneity (*I*^2^ = 65%; *P* = 0.003; Fig. 5). The funnel plot of studies assessing the association between BD and hypertension indicated no publication bias, which was supported by the results of the Begg’s test (*P* = 0.4042). Of these studies, two recorded systolic and diastolic blood pressure (DBP) levels in patients with BD [23, 24]. The pooled analysis showed no significant difference in systolic blood pressure (MD, 5.90; 95% CI −1.43–13.24; *P* = 0.11); however, an evident difference in DBP between patients with BD and HCs (MD, 3.73; 95% CI 1.20–6.27; *P* = 0.004) was observed (Supplementary Fig. 4).

### Behcet’s disease and obesity

The pooled analysis of ORs of two individual studies showed no significant association of BD with obesity (OR 1.09; 95% CI 0.26–4.49; *P* = 0.90; Supplementary Fig. 5A) [31, 35]. Thirteen case–control studies that included body mass index [22–29, 31, 34–36, 38], and six studies that included the waistline [24, 25, 27, 30, 33, 38] indicated no significant correlation between BD and body mass index (MD, 0.18; 95% CI −0.48–0.84; *P* = 0.59; Supplementary Fig. 5B), or between BD and waistline (MD, 0.77; 95% CI −1.33–2.88; *P* = 0.47; Supplementary Fig. 5C).

### Behcet’s disease and insulin resistance

Results for the analysis of the relationship between BD and insulin resistance are shown in Supplementary Fig. 6 [22, 26, 27, 29]. There was no obvious association between BD and insulin resistance (OR 2.12; 95% CI 0.75–6.03; *P* = 0.16), with substantial heterogeneity (*I*^2^ = 57%; *P* = 0.08; Supplementary Fig. 6). We conducted sensitivity analyses for BD and insulin resistance; the results did not show any significant alteration in the pooled OR when any individual study was sequentially omitted, demonstrating its stability and credibility. No evidence of potential publication bias was shown using Begg’s test (*P* = 0.4969). A subgroup analysis performed primarily based on different diagnostic tools of included trials revealed that patient with Behçet disease was associated with elevated incidence of insulin resistance when the diagnostic criteria for insulin resistance was hyperinsulinaemic-euglycaemic glucose clamp technique (OR 14.00; 95% CI 1.43–137.32; *P* = 0.02). But no obvious association between BD and insulin resistance was observed when the diagnostic criteria was the homeostasis model assessment of insulin resistance (HOMA-IR) formal score (OR 1.59; 95% CI 0.59–4.33; *P* = 0.36; Supplementary Fig. 7).

### The influences of additional factors

Besides potential immunologic interactions with metabolic processes, other factors including disease activity and treatment can also affect the risk of diabetes mellitus, arterial hypertension and dyslipidemia. In order to exclude the effect of drugs on the study, we excluded patients receiving drugs which may lead to metabolic complications such as steroids and DMARDs. The analyses showed similar results, and BD is significantly correlated with arterial hypertension (OR 1.26; 95% CI 1.07–1.48; *P* = 0.02; Supplementary Fig. 8A) and dyslipidemia (OR 1.14; 95% CI 1.05–1.24; *P* = 0.002; Supplementary Fig. 8B). Instead, the result indicated that there is no significant association between BD and diabetes mellitus (OR 1.20; 95% CI 0.94–1.53; *P* = 0.14; Supplementary Fig. 8C).

## Discussion

**Fig. 6:**
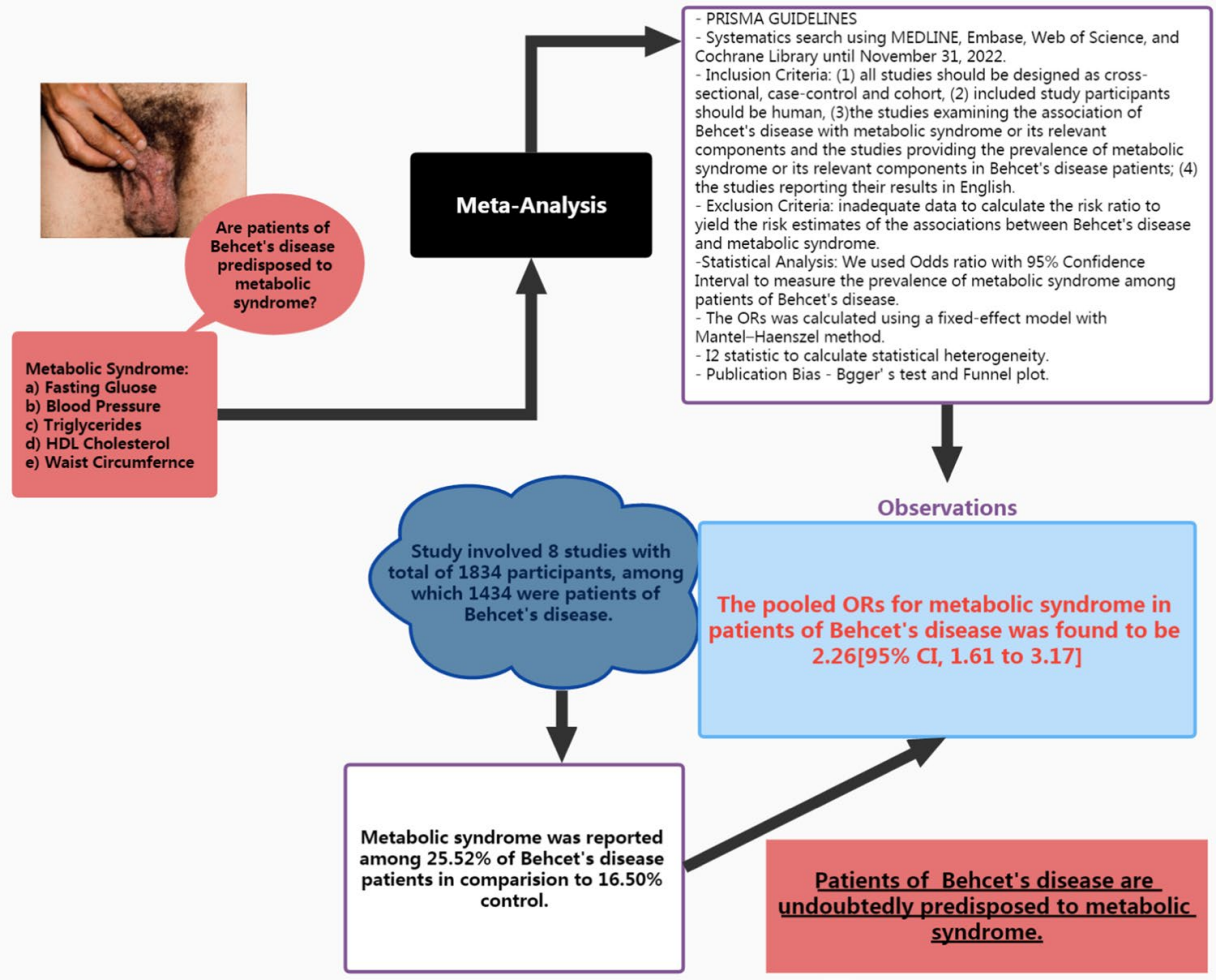
Outline to assess methodology and key observations of Behçet’s disease and its association with metabolic syndrome

This meta-analysis quantitatively investigated the relationship between BD and the risk of MetS and its components. Twenty-three observational studies were included to investigate the prevalence of MS and its components in patients with BD [14, 22–37]. In total, 9,686 patients with BD and 26,926 controls were enrolled in our analysis. The cumulative assessment of this meta-analysis indicates that MetS has emerged as an important associative factor in patients with BD (Fig. 6). Individual risk factors for diabetes mellitus, hypertension, and dyslipidemia are linked to comorbidities in patients with BD.

Consistent with previous reports, our study demonstrated a prominent association between BD and MetS. In the present study, the risk of MetS in patients with BD was 126% higher than in the healthy control group (OR 2.26; 95% CI 1.61–3.17; *P* < 0.0001). Furthermore, there was no significant heterogeneity or publication bias in the analysis. Further, the results of sensitivity analysis were stable and credible. Our findings revealed that BD is significantly associated with insulin resistance, diabetes mellitus and hypertension. Moreover, based on all the included studies, the association of BD with fasting blood glucose (a diabetes-associated parameter) and systolic blood pressure (a hypertension-associated parameter) was not statistically significant. However, there was an evident difference in DBP levels between patients with BD and non-BD controls; therefore, the DBP probability is a potential marker for predicting the incidence of BD-related hypertension. Still, the number of included studies reporting DBP levels was limited, and more studies are needed to confirm this result. In addition, when we excluded the effect of medications, the relationship between BD and diabetes was not statistically significant. Therefore, physicians should pay attention to the distinction between complications and drug effects in order to facilitate further treatment of patients.

Additionally, although substantial heterogeneity was observed across the included studies, a significant association between dyslipidemia and BD was reported in our study, and decreased HDL levels in patients with BD were observed in the pooled analysis. A previous study reported that low HDL levels were closely related to the dysfunction and inflammation of vascular in patients with BD; however, no significant association between BD and HDL level have been reported in our study. Further observational studies are urgently needed to analyze the relationship between HDL levels and BD.

MetS is a clinical condition characterized by a series of metabolic risk factors [11], and is an important risk factor for cardiovascular diseases [11]. BD can be described as a multifactorial disease that may also affect the cardiovascular system [42]. The association of BD with MetS, diabetes mellitus, hypertension, and dyslipidemia potentially arise from a similar etiopathogenesis between BD and these metabolic disorders. Inflammation plays a vital role in BD, and elevated circulatory proinflammatory cytokines—including interleukin-1, interleukin-6, interleukin-8, and tumor necrosis factor-alpha (TNF-α)—can be found in patients with BD [9, 43]. These inflammatory markers also cause the downregulation of insulin activity, which leads to insulin resistance, endothelial dysfunction, and the development of MetS [44]. TNF-α has also been associated with the pathogenesis of insulin resistance and diabetes mellitus [45, 46]. Regarding immune disorders, the dysfunction of regulatory T cells also regulates the development of both BD and hypertension [43, 47]. Moreover, BD and MetS negatively impact patients’ quality of life and psychological health [48, 49]. The inflammatory process of BD and MetS may explain the psychological damage. Moreover, the level of inflammation in vivo can also be enhanced by emotional stress, and psychological factors play an essential role in the development of BD and MetS.

### Limitation

Given the limited number of studies assessing the prevalence of MetS or its different components in patients with BD and diverse clinical characteristics, no data were available to perform subgroup analyses based on the type, severity, or activity of BD. In addition, the inclusion of patients in this study was limited to few regions and included various study designs.

### Conclusion

In summary, our systematic review and meta-analysis suggests that patients with BD (25.52%) are more predisposed to MetS than the general population (16.50%). An association between BD and the risk of MetS components—including diabetes mellitus, dyslipidemia, and hypertension—was also identified. Based on these findings, we recommend that physician consider these associations so that specific attention and treatment are available for patients with particular comorbidities. We also advise that patients with BD regularly monitor their blood pressure, triglyceride, fasting plasma glucose, and HDL cholesterol levels, as well as waist circumference.

### Supplementary Information

Below is the link to the electronic supplementary material.

Supplementary file1 (DOCX 1252 KB)

## Data Availability

Data availability is not applicable to this article as no new data were created or analyzed in this study.
