# Supplementary material for: Association of Behçet’s disease with the risk of metabolic syndrome and its components: a systematic review and meta-analysis
Source: Clin Exp Med. 2023 Mar 20;23(6):2855–66. doi: 10.1007/s10238-023-01044-x (PMC10543763; doi:10.1007/s10238-023-01044-x)
Supplement: Supplementary file 1 — Supplementary file1 (DOCX 1252 KB) [file 10238_2023_1044_MOESM1_ESM.docx]

Supplementary Material

Association of Behçet’s Disease with the Risk of Metabolic Syndrome and its Components: A Systematic Review and Meta-analysis

Tingqiao Chen^1†^, Xinyi Shao^1†^, Hao Li^2^, Yangmei Chen^1^, Lin Liu^1^, Judan Zhong^1^, Jin Chen^1*^

*** Correspondence:** Jin Chen: chenjin7791@163.com

# Supplementary Figures

**Supplementary Figure 1.** Forest plot for the association between Behçet’s disease and fasting blood glucose level.
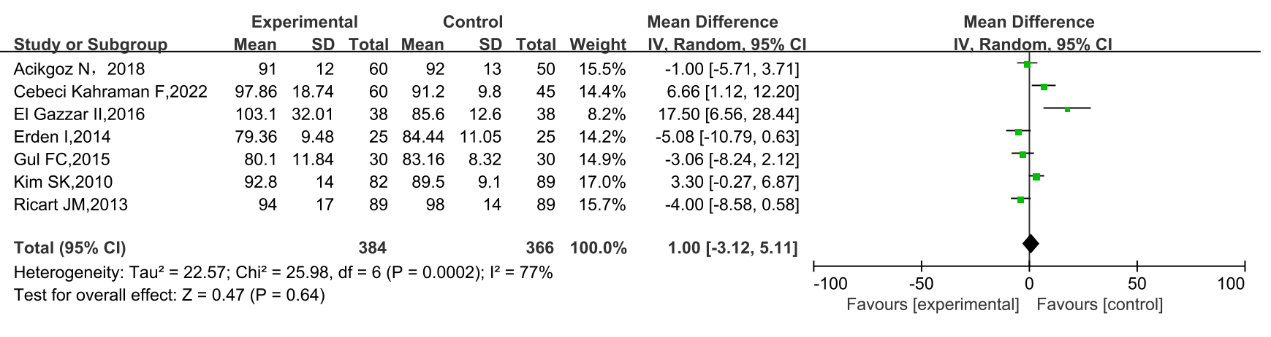


**Supplementary Figure 2.** Forest plot for the association between Behçet’s disease and triglyceride level.


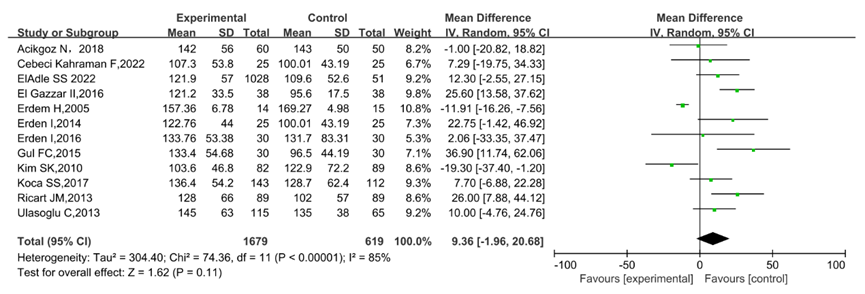


**Supplementary Figure 3.** Forest plot for the association between Behçet’s disease and high-density lipoprotein level.


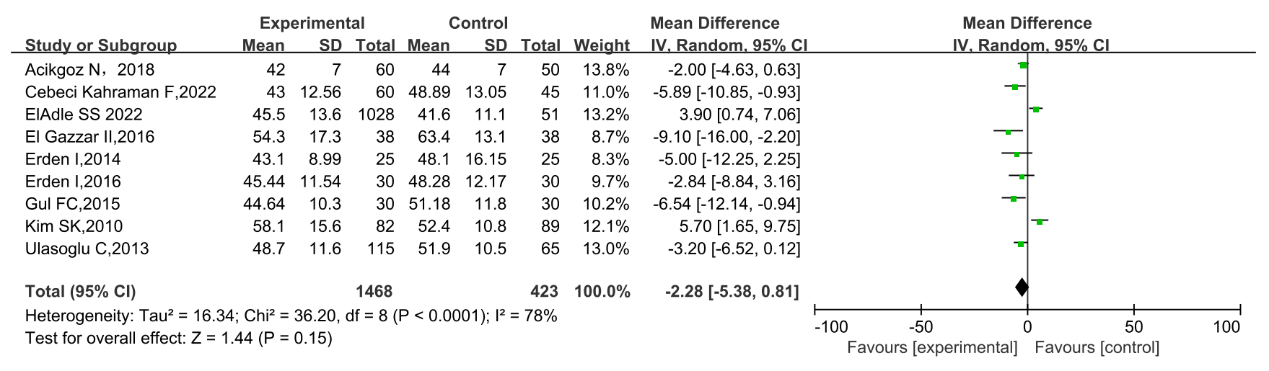


**Supplementary Figure 4.** Forest plot for the association between Behçet’s disease and diastolic blood pressure.


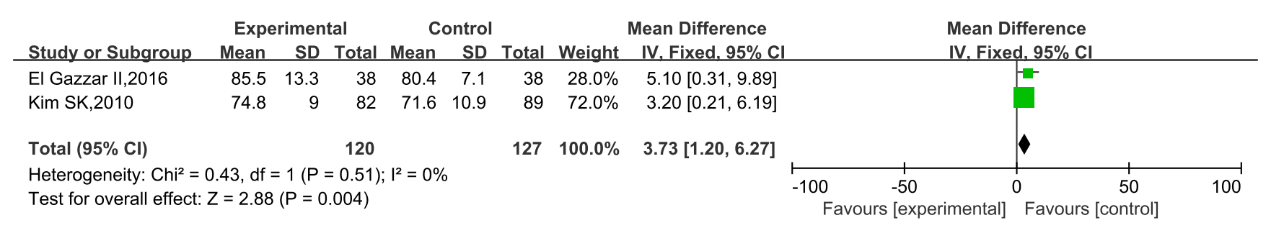


**Supplementary Figure 5.** Forest plot for the association between Behçet’s disease and obesity **(A)**. Forest plot for the association between Behçet’s disease and body mass index **(B)**. Forest plot for the association between Behçet’s disease and waistline **(C)**.


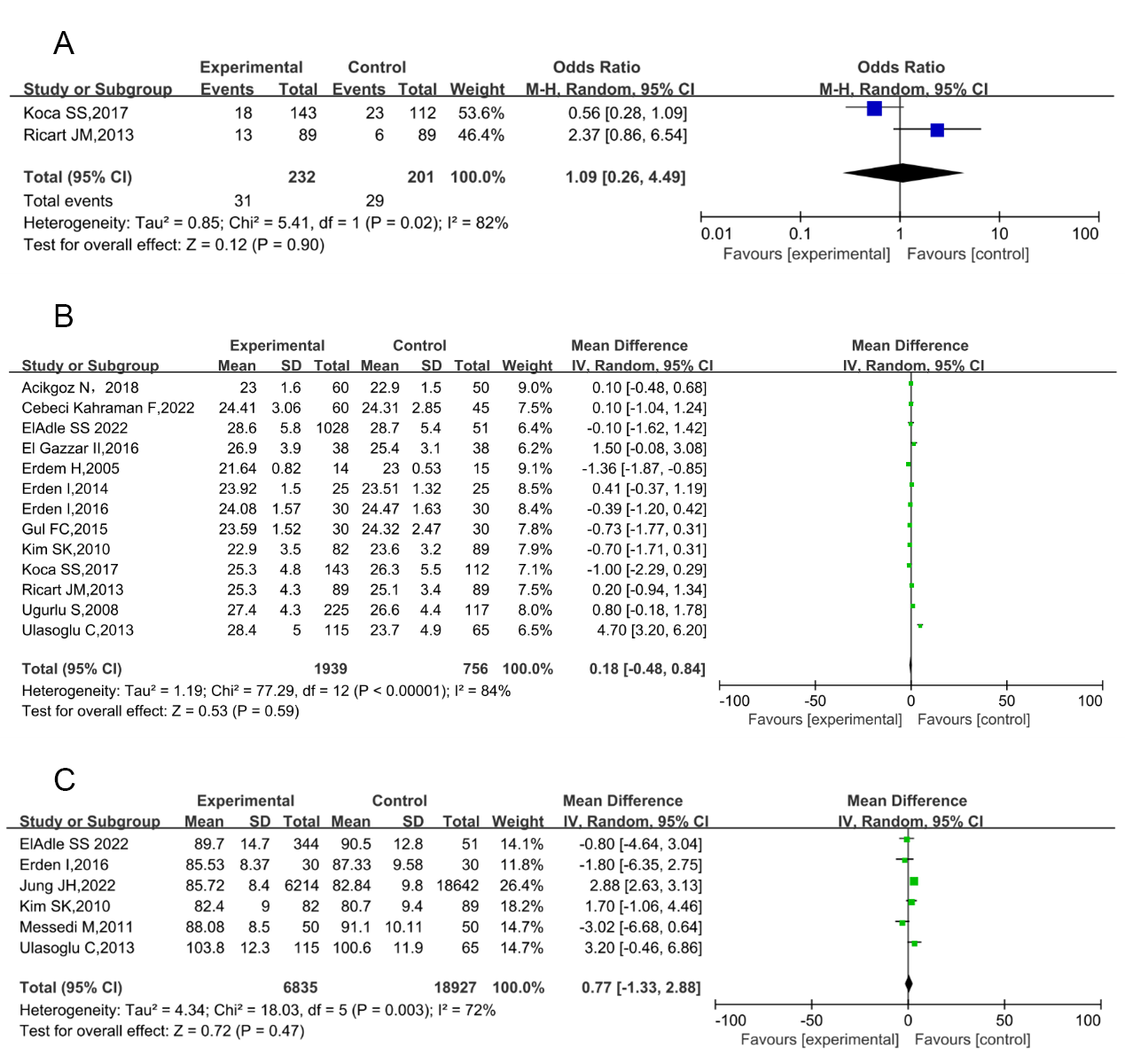


**Supplementary Figure 6.** Forest plot for the association between Behçet’s disease and insulin resistance.


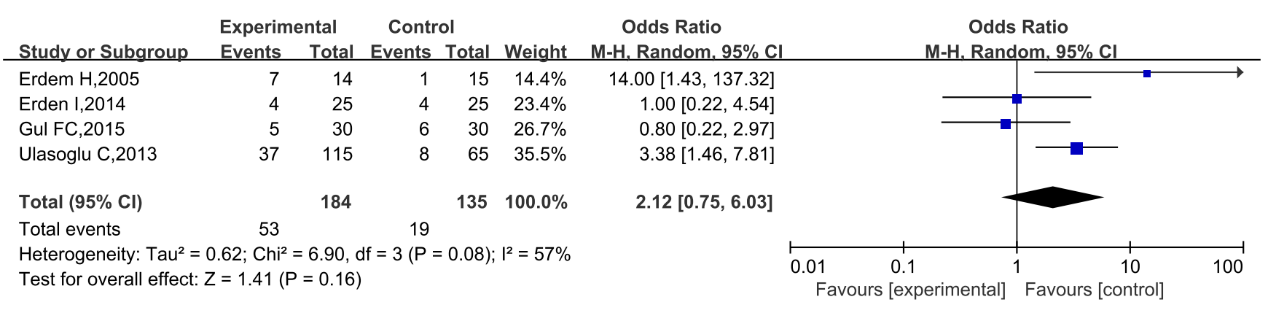


**Supplementary Figure 7.** Forest plot for the Subgroup analysis between Behçet’s disease and insulin resistance.

**
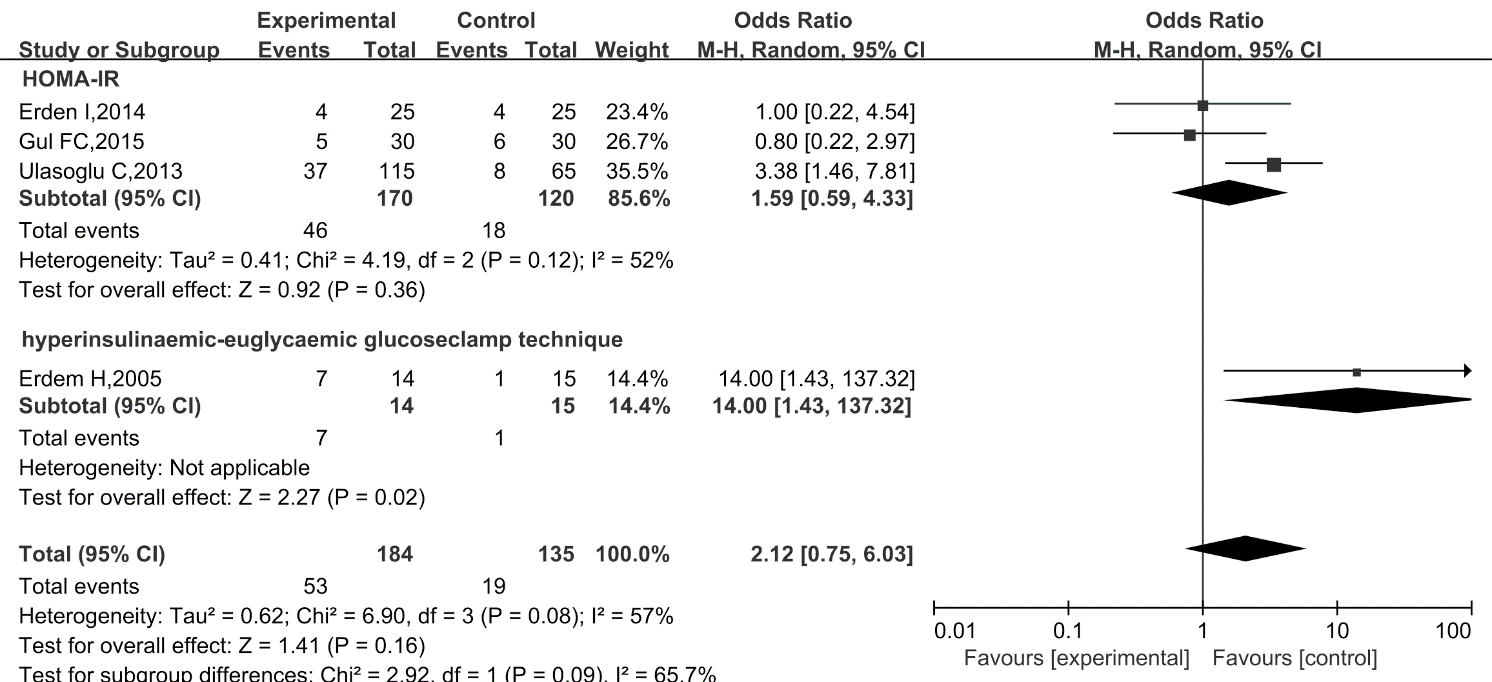
**

**Supplementary Figure 8.** Analyses performed after exclusion of drug effects. Forest plot for the association between Behçet’s disease and hypertension **(A)**. Forest plot for the association between Behçet’s disease and dyslipidemia **(B)**. Forest plot for the association between Behçet’s disease and diabetes mellitus **(C)**.

**
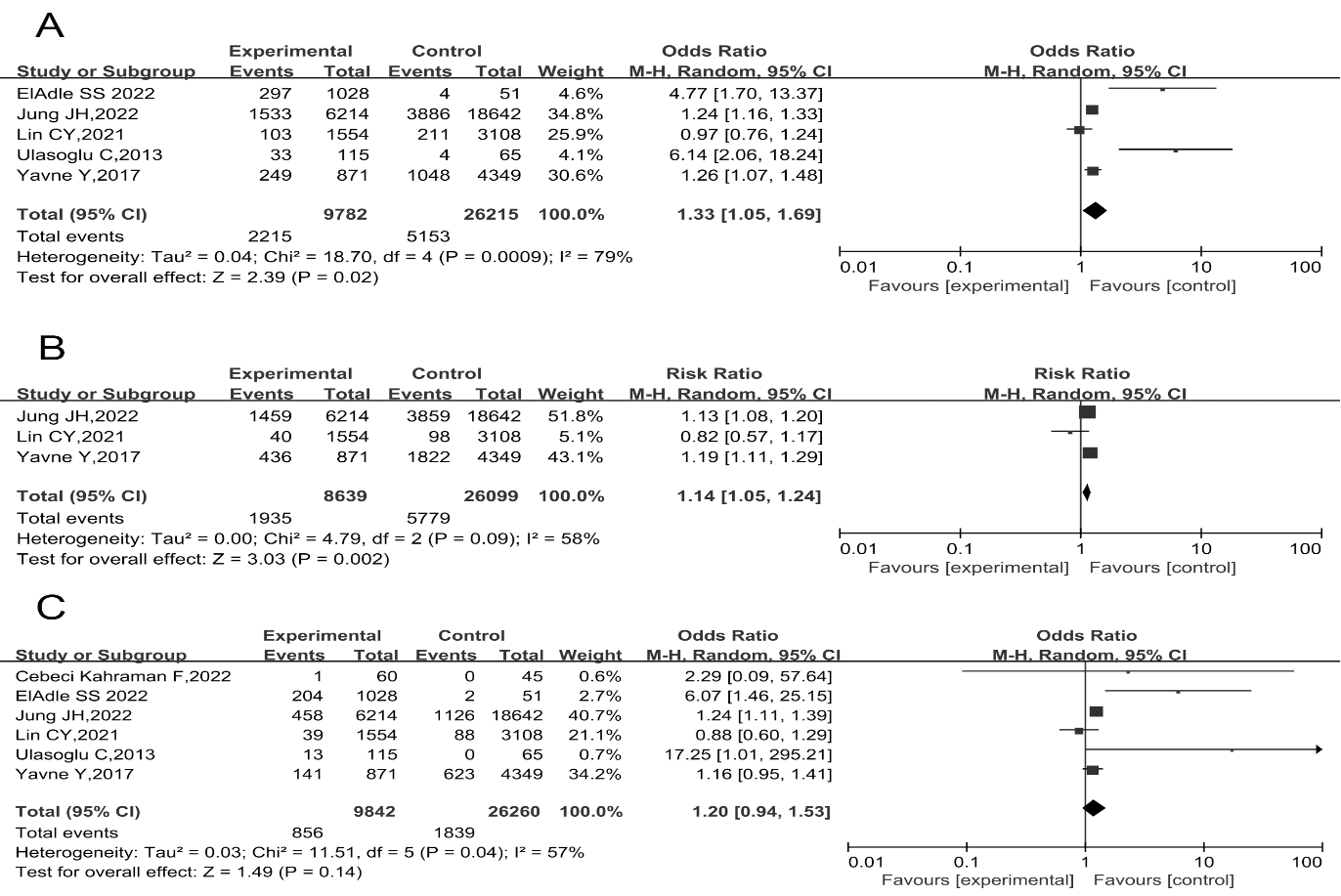
**
